# Study of the Influence of Sociodemographic and Lifestyle Factors on Consumption of Dairy Products: Preliminary Study in Portugal and Brazil

**DOI:** 10.3390/foods9121775

**Published:** 2020-11-30

**Authors:** Raquel P. F. Guiné, Sofia G. Florença, Solange Carpes, Ofélia Anjos

**Affiliations:** 1 CERNAS Research Centre, Polytechnic Institute of Viseu, 3504-510 Viseu, Portugal; raquelguine@esav.ipv.pt; 2 Faculty of Food and Nutrition Sciences, University of Porto, 4200-465 Porto, Portugal; 3 Department of Chemistry, Federal University of Technology—Paraná (UTFPR), 85503-390 Pato Branco, Brazil; carpes@utfpr.edu.br; 4 Polytechnic Institute of Castelo Branco, 6001-909 Castelo Branco, Portugal; ofelia@ipcb.pt; 5 Forest Research Centre, School of Agriculture, University of Lisbon, 1349-017 Lisbon, Portugal

**Keywords:** milk, cheese, butter, yogurt, questionnaire survey

## Abstract

Sociodemographic characteristics, including regional variations, have been associated with different food consumption patterns. Behavioral factors and lifestyle variables may also contribute to different food dietary trends. In this way, the present study intended to investigate the consumption habits of the most relevant types of dairy products around the world and relate them to sociodemographic factors, for example, age, sex, education and country as well as with some anthropometric and behavioral aspects, for example, body mass index, satisfaction with body weight and exercise or sedentary lifestyles. One other objective of the study was to categorize the lifestyles of the participants, according to measured variables linked with hours of inactivity or exercise, in order to use these as possible differentiating variables for the consumption of dairy products. The study involved a questionnaire survey undertaken on a non-probabilistic convenience sample of participants from Portugal (PT) and Brazil (BR), and participation was voluntary and anonymous. The data analysis involved different statistical techniques: basic statistics, chi-square tests, factor analysis, cluster analysis and tree classification analysis. The results showed that semi skimmed milk is never consumed by about half of the participants (47.4% for PT and 46.7 for BR), and those numbers increase for skimmed (64.8% for PT and 50.9% for BR), chocolate flavored milk (82.6% for PT and 65.6% for BR) and enriched milks (94.8% for PT and 85.3% for BR). Cheeses are also consumed in the two countries by small numbers of people. The number of participants consuming imported cheeses in both countries was particularly low (only 4.0% consume these more than once a week in both countries), suggesting national products may be preferred. It was further observed that those who consume cheese do it seldom (once a week) or sometimes (2–3 times per week). Butter is also consumed by only about half of the adult population (43.8% for PT and 49.5% for BR), but the percentage of those who never consume butter increases for skimmed butter (66.0% for PT and 82.6% for BR) and unsalted butter (70.2% for PT and 69.1% for BR). The consumption of yogurts also follows similar low consumption patterns. The most frequently consumed yogurt types in Portugal are liquid (30.5% consume regularly) and natural yogurts (34.8% consume regularly), while in Brazil the most frequent are creamy fruit pulp yogurt (14.4% consume regularly), liquid (13.7% consume regularly) and Greek type yogurt (10.2% consume regularly). A factor analysis and a cluster analysis established groups according to lifestyles, as follows: 1—Screeners, 2—Exercisers, 3—Travelers and 4—Others. These lifestyles were found to be influential in the consumption of dairy products for all classes of dairy tested: milk, cheese, yogurt and butter. For example, the screeners were found to consume more milk, more butter, more cheese and more yogurt. Additionally, other influential factors were age, sex, education, BMI and satisfaction with body weight. Nevertheless, country was not a meaningfully discriminant variable in relation to the other variables included in the classification analysis. The results concluded that, despite some small differences in the patterns of consumption of dairy products in both countries, the levels of consumption of dairy products are extremely low, for all classes studied (milk, cheese, yogurt or butter). Additionally, it was concluded that some factors are influential on the level of consumption of dairy products, and therefore decision makers can plan their interventions according to the characteristics of the targeted segments of the population, according to lifestyle, age, sex, education, BMI and satisfaction with body weight.

## 1. Introduction

Dairy products are one of the basic food categories in human diets all over the world. These food products include milk and its derivatives, for example, butter, cheese, fermented milk and yoghurts. According to the Milk Market Observatory of the European Commission in October 2020 [1], in the European Union in the last 12 months there was an increase of 1.6% in the collection of cow’s milk, a small decrease in the production of fermented milk by 0.6%, an increase of 1.4% in the production of cheese and an increase of 2.2% in the production of butter. The highest increase was observed for powdered whole milk at 5.6%. Regarding yogurt production in the 28 countries of the European Union, in 2019 8163 thousand tons of acidified milk products were produced, including yogurts and others [2]. Data reporting to the World production of the main dairy products issued by the Milk Market Observatory of the European Commission released in May 2020 [3] show that in the United States in 2019 5959 thousand tons of cheese and 904 thousand tons of butter were produced. The data further shows that the primary milk producer in 2019 was India (194,200 thousand tons), followed by the European Union (160,000 thousand tons) and the United States (99,155 thousand tons).

The consumption of dairy products is recommended by many organizations around the world due to their nutritional richness, and for that reason they are indicated for consumption throughout the entire life cycle, from infants to elderly [4,5].

Dairy products can contain high amounts of protein of high biological value, as well as vitamins (particularly the fat-soluble vitamins A and D and the B complex vitamins B_2_—riboflavin and B_12_—cobalamin) [6,7,8,9] and dietary minerals (such as calcium, phosphorus, sodium, magnesium, potassium and iodine) [10,11,12]. They also contain carbohydrates, especially in the form of lactose, which might be problematic for some consumers with lactose intolerance. However, people suffering from this pathology, which causes gastrointestinal malfunctioning, do not derive their symptoms exclusively form the consumption of dairy products, contrarily to popular belief, as discussed by Jansson-Knodell et al. [13]. Usual gastrointestinal symptoms that can appear after lactose ingestion in individuals with lactose intolerance include abdominal pain, diarrhea, flatulence and bloating [14]. Some people believe they have lactose intolerance when they in fact have not been diagnosed by a physician, and their gastrointestinal problems are derived from something other than lactose [13,15]. Nevertheless, those individuals who believe themselves to be lactose intolerant or who follow fashionable dietary trends tend to avoid milk and other related dairy products [15,16,17]. By avoiding the consumption of dairy products these individuals may suffer from insufficient basic nutrients, increasing the risk of certain health problems, for example, poor bone health, in turn increasing the risk for osteoporosis [14,18]. Besides, dairy products contain fat, part of which is saturated, a type of fat that has been associated with some health problems. However, this is not consensual because there are a large number of studies which have proven the contrary, i.e., that limiting dietary saturated fatty acid intake showed no benefits on cardiovascular disease and total mortality, while showing protective effects against stroke and high blood pressure [19,20]. Finally, dairy products also contain substances with physiologically active properties, such as antioxidants, oligosaccharides, probiotic bacteria, butyric acid, immunoglobulins and active peptides, which have been investigated for their beneficial roles in the human body [21,22,23,24,25].

Sociodemographic characteristics are usually associated with different food consumption patterns. Additionally, behavioral factors and lifestyle variables also contribute to different food dietary trends. For example, Khandpur et al. [26] found differences in the consumption of ultra-processed foods according to age, socioeconomic status, area of residence and geographical region but not gender. Riedeiger et al. [27] reported that female gender, household education and high income had a positive impact on fruit and vegetable consumption among adolescents. Nayga [28] reported sociodemographic influences on consumer concern about irradiated foods, or foods containing antibiotics, hormones and pesticides.

In the case of dairy products, Richter and Sanders [29] evaluated sociodemographic influences on the consumption of dairy products in Switzerland and concluded that age and income were important influencers for consumption of organically produced dairy products. Miftari et al. [30] investigated the role of demographic and socioeconomic factors on dairy product consumption in Kosovo and found that age, education, income, household size and number of children influenced consumption patterns. Shi et al. [31] reported that socio-economic status was strongly associated with consumption of milk in Chinese adolescents. Baek and Chitekwe [32] observed differences in the consumption of diverse foods, including dairy products, according to geographic location and income.

The consumption of dairy products may have a preventive role in some diseases that affect people all over the world, and therefore in Portugal and Brazil. One of the most recognized health benefits associated with the consumption of dairy products is the prevention of osteoporosis [33,34,35]. The study by Rodrigues et al. [36] aimed at characterizing the state of osteoporosis in post-menopausal women in Portugal and showed that 43% of them suffered from osteoporosis, leading to important healthcare consumption and treatment costs. In Brazil, according to Radominski et al. [37], the guidelines issued by the Osteoporosis and Osteo-metabolic Diseases Committee of the Brazilian Society of Rheumatology, in conjunction with the Brazilian Medical Association and other Societies, dictate that women over 50 should ingest up to 1200 mg of calcium per day, preferably in their diet, by consuming milk and dairy products. As far as we know, the consumption of dairy products in Portugal and Brazil have not been investigated, and therefore, this study was carried out to investigate people’s consumption of dairy products and to what extent sociodemographic, anthropometric and behavioral factors associated with lifestyle influence the consumption of dairy products. Additionally, and because the study was carried out in two countries, differences between the countries were investigated. One other objective of the study was to categorize the lifestyles of the participants, according to measured variables linked with hours of inactivity or exercise, for example, in order to use these as possible differentiating variables for the consumption of dairy products.

## 2. Materials and Methods

### 2.1. Data Collection and Sample Characterization

This work was based on the application of a questionnaire, which was developed purposely for this research, in order to investigate consumption habits regarding dairy products, such as milk, yogurt, cheese or butter. The questionnaire was validated and approved for the study. The questionnaire included different parts, as follows: Part 1: Sociodemographic data (5 questions); Part II: Anthropometric and behavioral aspects (10 questions); Part III: Consumption habits regarding dairy products (21 questions distributed as follows—4 for milk products, 7 for cheeses, 3 for butter, 7 for yogurt). The complete questionnaire is attached in Appendix A. The data collected was based on the participants’ self-responses for frequencies of consumption on the scale provided: Never, Seldom (Once/week), Sometimes (2–3 times/week), Frequently (once/day) and Always (+ 1 time/day).

To undertake this descriptive cross-sectional study a non-probabilistic convenience sample was used, consisting of adult citizens from Brazil and Portugal. Hence only participants aged 18 years or older were included in the study, and these had to provide consent to participate. One other inclusion criteria, derived from the mode of survey delivery, was to have access to a computer and internet as well as to have the necessary knowledge to be able to use the technology and the platform through which the questionnaire was presented. These two countries were selected due to being connected through language and culture, since Brazil was once a colony of Portugal, and also because nowadays they receive different influences related to geographical location: one in the South American continent and the other in Western Europe. The choice of a convenience sample was owing to facility of recruitment and place of residence, and it was intended to have a similar sample size in each of the countries. One important advantage of convenience samples is precisely the ease of recruitment, despite not allowing generalization according to estimates of sociodemographic differences. Still, they represent an adequate tool for exploratory research, as is the present case [38,39,40,41,42]. The survey took place through an internet questionnaire platform and 914 answers were obtained. The data collection took place between November 2019 and April 2020. The answers were obtained only from adult citizens (aged 18 or over), and after informed consent. Extreme care was taken to verify all ethical guidelines when formulating and applying the questionnaire. Finally, confidentiality of the individual answers was guaranteed to comply with ethical principles. The survey was registered and approved by the Ethics Committee of the Polytechnic Institute of Viseu before application.

### 2.2. Data Analysis

Body mass index (BMI) was calculated from self-reported values of height and weight, according to the equation:

(1) $$BMI\;\left(kg/m^{2}\right)\;=\;\frac{Weight\;\left(kg\right)}{{\left[Height\;\left(m\right)\right]}^{2}}$$

Participants were then classified according to the World Health Organization (WHO) classification for BMI as: underweight: BMI < 18.5; normal weight: 18.5 ≤ BMI < 25; overweight: 25 ≤ BMI < 30; obesity: BMI ≥ 30. There are other classes of obesity, differentiating the degree of obesity, but for the present study these four classes were considered sufficient [43,44].

Sociodemographic information was also collected and age was classified into categories according to: young adults (aged between 18 and 30 years), middle aged adults (between 31 and 50 years), senior adults (between 51 and 65 years), and the elderly (aged 66 years or over).

For the analysis of the data different basic descriptive statistical tools were used, such as frequencies and descriptives, including minimum, maximum, mean value and standard deviation. The crosstabs and the chi square test were used to access the relations between some of the categorical variables under study. Moreover, the Cramer’s V coefficient was used to analyze the strength of the significant relations found between some of the variables. This coefficient ranges from 0 to 1 and can be interpreted as follows: V ≈ 0.1, the association is considered weak, V ≈ 0.3, the association is moderate, and V ≈ 0.5 or over, the association is strong [45].

Factor analysis (FA) and cluster analysis (CA) were used to treat data obtained for lifestyle variables: •Practice of physical exercise; ∙Daily hours traveling walking or riding a bike; •Daily hours watching TV; •Daily hours playing with computer or mobile phone or on social networks; •Daily hours working on computer; •Daily hours traveling inactively (in car, motorbike, bus, train, etc.). The correlation matrix, the Kaiser-Meyer-Olkin measure of adequacy of the sample (KMO) and Bartlett’s test were all used to confirm the adequacy of data for FA [46]. The FA was applied with extraction by principal component analysis (PCA) method and Varimax rotation with Kaiser Normalization, being the number of components determined by the Kaiser criterion (eigenvalues ≥1) and also by the scree plot. Communalities were calculated to show the percentage of variance explained by the factors extracted [46]. Only factor loadings with absolute of 0.4 or higher were used [47,48].

Cluster analysis (CA) of the factors resulting from FA started with four hierarchical methods to find the most adequate number of clusters, based on the agglomeration schedule: average linkage—between groups (AL-BG), average linkage—within groups (AL-WG), centroid and Ward. This procedure allowed fixing the number of clusters at four and then applying the k-means, which is particularly recommended and frequently used in cluster analysis [49]. The application of k-means was made using the initial solutions obtained with the four hierarchical methods.

To treat the data obtained for the frequency of consumption of certain dairy products, a mean value was computed for each of the dairy product categories: milk, cheese, butter and yogurt. The measurement scale for the individual consumption variables was: 1—Never, 2—Seldom (once/week), 3—Sometimes (2–3 times/week), 4—Frequently (once/day) and 5—Always (more than once/day). However, the variables accounting for the average consumption for each category were real numbers varying from 1 to 5, and their interpretation was made according to the following defined scale: low consumption—value ∈ (1.0, 2.5), moderate consumption—value ∈ (2.5, 3.5), high consumption—value ∈ (3.5, 5).

The variables accounting for the average level of consumption of the four categories of dairy products were submitted to a tree classification analysis for evaluation of the relative importance of each of the possible influential variables considered: country, sex, age class, education, BMI class, satisfaction with body weight, balanced diet, and lifestyle clusters. The analysis followed the CRT (Classification and Regression Trees) algorithm with cross validation and with minimum change in improvement of 0.0001, considering a limit of 5 levels and the minimum number of cases for parent or child nodes equal to 30 and 20, respectively.

The data were processed using the SPSS program, version 26 from IBM, Inc, and the level of significance used was 5%.

## 3. Results and Discussion

### 3.1. Sociodemographic Characterization of the Sample

From the 914 participations obtained, 64 were rejected because the questionnaires were incomplete or not properly filled out, resulting in a sample of 850 valid responses, 430 from Brazil and 420 from Portugal. Table 1 shows the sociodemographic characteristics of the 850 participants included in the sample studied. The number of participants from both countries was similar, corresponding to 50.6% from Brazil and 49.4% from Portugal, but the distribution according to sex was not equal, having more women (65.1%) than men (34.9%).

The participants were aged between 18 and 82, most of them, 47.5%, were middle aged adults (26 ≤ age ≤ 50 years), and those less represented, only 1.4%, were the elderly (age ≥ 66 years). The distribution of the participants according to their marital status shows that most of them were married (48.5%) or single (43.8%). Regarding the level of education, the large majority of the participants had completed a university degree (86.7%).

### 3.2. Anthropometric Measures

Table 2 presents the anthropometric measures of the participants. The average height was 1.68 ± 0.09 m for the whole sample, with men showing higher values (1.76 ± 0.07) as compared with women (1.63 ± 0.06), as usually happens. The average weight was 70.06 ± 15.80 kg, and the difference between men and women was considerable for this sample: 63.71 ± 11.96 and 81.87 ± 15.28 kg, respectively. The calculated BMI was 24.73 ± 4.50 kg/m^2^ for the whole sample, being also higher for the men (26.34 ± 4.40 kg/m^2^) as compared to women (23.86 ± 4.31 kg/m^2^). It is important to notice that these measures were self-reported and, therefore, some inaccuracy might be present, this being a limitation of this kind of assessment of anthropometric measures.

The participants’ BMI was classified according to the World Health Organization into the four principal classes as shown in Table 3. Most participants have a normal weight (56.5% for the whole sample), but mostly in women (65.0%) when compared with men (40.4%). However, the incidence of overweight is worrying (26.0% for the whole sample), particularly in men (39.1%), while in women the percentage is lower (19.0%), but still important. Even raising more concern is the percentage of obese participants (13.4%, 18.5% and 10.7%, respectively for global, men and women), considering the risks that this brings to health, associated with diabetes and heart related diseases [50,51,52,53].

### 3.3. Lifestyle

One aspect investigated related to the participant’s self-perception about the practice of a balanced diet. Unbalanced diets lead to malnutrition, referring either to under-nutrition or to over nutrition. Nevertheless, very often malnutrition is associated with excessive calories in relation to requirements, coupled with low intake of micronutrient-rich foods [54]. Food security entails aspects such as availability, utilization and access, but these may not necessarily cover nutritional aspects. In fact, easily accessed and available food products may not necessarily provide a healthy and balanced diet. The Family Nutrition Guide of the FAO (Food and Agriculture Organization of the United Nations) [55] states that a balanced diet should be able to provide the right amount of food, energy and nutrients needed during the day to cover the dietary requirements of a particular person. Additionally, a balanced diet must include a variety of foods from different food groups to ensure the supply of all the nutrients needed for adequate body functioning, thus significantly contributing to a healthy life, while at the same time lowering mortality incidences related to malnutrition. Because a healthy diet needs to be balanced, the participants were asked about the frequency with which they practiced a balanced diet.

Table 4 shows the results obtained for this question, considering the possible effect of some sociodemographic variables, assessed through chi-square tests. When considering the global sample, it was found that nearly half of the participants (46.9%) believed they frequently practice a balanced diet, while 16.5% responded that they always have a balanced diet. A considerable fraction (30.0%) responded to eating a balanced diet only sometimes. 

Additionally, Table 4 also shows that no significant differences were found between sexes (*p* = 0.060), but considering the two countries involved significant differences were encountered (*p* < 0.0005), with participants from Portugal reporting a higher frequency of balanced diet as compared with Brazilians, although the association between these two variables (balanced diet and country) was weak (V = 0.198).The results in Table 4 also show that high percentages of underweight and obese participants admitted to practicing a balanced diet only sometimes (45.7% and 37.7%, respectively), and people who are overweight believed they frequently consume a balanced diet (50.7%). These differences were significant (*p* = 0.001), but with a low association (V = 0.116). Considering age classes, an significant number of young adults state that they have a balanced diet only sometimes (41.6%), but the care for a balanced diet increases for the elderly, with 58.3% saying that they always have a balanced diet. These differences were significant (*p* < 0.0005). Older people tend to have more health problems, sometimes suffering from non-communicable and age related diseases, thus making it more important to have a healthier food intake, either because they want to remain healthy or to diminish the morbidity associated with their diseases. Results from countless studies have revealed that healthy dietary patterns are associated with a reduced risk of developing several chronic diseases or having a better control of these, such as obesity, dyslipidemia, hypertension and diabetes mellitus. Hence, for the elderly, nutrition assumes a particular role in the maintenance of acceptable health standards and functional capacity [56,57]. As for marital status, the widowed showed a higher frequency of balanced diet as compared with the other groups (single, married or divorced), with 61.5% having a balanced diet frequently and 15.4% always. These results are in line with those of age class, since most often the widowed consisted of older people, who had already lost their spouses. The differences are significant (*p* = 0.009), although the association between balanced diet and marital status is weak (V = 0.102). Finally, the results showed that higher education levels are associated with higher frequency of practicing a balanced diet, with the percentages of participants who report this frequently increasing systematically from 20.0% for people with the lowest level of education completed, up to 47.8% for participants with a university degree. Again, these differences were significant (*p* < 0.0005). These results clearly indicate that more educated people are better aware of the importance given to a proper diet to benefit from a globally healthier status [58].

Another aspect investigated in this research was the satisfaction with body weight, and the results are shown in Table 5 for the global sample and also according to BMI classes and to the practice of a balanced diet. Most participants (40.1%) believed they are of normal weight but would still like to lose 2 to 5 kg.

Considering the underweight participants, 42.9% are aware of their condition, and stated they would like to gain a few kilos (Table 5). Of the participants with normal weight, an important part (39.5%) is satisfied with their body weight and wish to maintain it, while 46.4% would like to lose 2 to 5 kg, even though they know their weight is normal. For those participants who are overweight, a high percentage (48.9%) wrongly think they have normal weight but would still like to lose 2 to 5 kg, and 32.1% are conscientious about being overweight. From the obese participants, 75.4% know about their condition and would like to lose a few kilos, but say they have tried and not succeeded. These results are interesting, because they reveal that, in general, the participants were aware of their bodyweight status.

The results in Table 6 refer to the practice of physical exercise and also the level of activity related to active daily traveling, by walking or riding a bike, for example. Most participants (38.8%) practiced exercise occasionally, i.e., once a week, but a considerable part (33.9%) does 2 to 3 times a week, which is the frequency recommended for most cases. Nevertheless, 17.4% of participants never do physical exercise, which can have a very negative impact on health at so many levels [59,60,61,62]. Regarding active traveling, for example to and from work, most participants (52.2%) do not or do less than half an hour per day, but 33.8% spend between 30 min and 1 h daily in these activities.

Table 7 presents the results obtained for the hours spent daily on sedentary activities. The results indicated that a high percentage (32.4%) spend between 1 and 2 h watching TV, and 13.8% spend up to 5 h watching TV. The participants do not engage much in activities like playing with computers or mobile phones or on social networks, i.e., screen entertainment, with 39.9% spending less than 30 min per day in those activities. On the other hand, the use of computers for professional purposes is a reality for the great majority of the participants, with 30.4% spending between 2 to 5 h, and 39.6% spending more than 5 h daily working on the computer. Finally, the daily hours of inactive traveling, for example in a car, motorbike, bus or train, are low, less than 30 min for 54.2% of participants, or between 30 min and 1 h for 30.4% of participants.

#### 3.3.1. Factor Analysis for Lifestyle

Some variables related to lifestyle were subjected to exploratory FA, starting with Principal Component Analysis (PCA), to identify a possible grouping structure between the variables used to evaluate aspects related to physical activity or sedentary lifestyle of the participants.

The correlation matrix showed some correlations between the variables, although they were relatively weak. The value of KMO was low (0.496), but the results of the Bartlett’s test of sphericity indicated adequacy for applying FA (*p* < 0.0005), thus leading to the rejection of the null hypothesis that the correlation matrix was equal to the identity matrix. Analysis of the anti-image matrix revealed that values of MSA (Measure of Sampling Adequacy) were close or above 0.5, meaning that in general the variables could be included in the analysis. The solution obtained by rotation of FA with PCA originated three components, explaining 59.7% of total variance, distributed by the three factors as: F1—22.4%, F2—19.3% and F3—18.0%. All variables had communalities higher than 0.4: the variable practice of physical exercise had the highest value (0.827, indicating that this variable had 82.7% of its variance explained by the solution), while the variable with lowest communality was daily hours working on computer (0.509). Rotation converged in five iterations and extracted three factors, grouping the variables as shown in Table 8.

Considering the components in Table 8, F1 was clearly linked to activities related to screens, where watching TV and playing on screen devices have positive high loads, while the use of computer for work has a considerable load, but negative, indicating that these variables contribute strongly to the definition of the factor. Factor F2 is strongly linked with variables related to daily hours of traveling, either active or inactive, and finally F3 is very strongly related with only one variable, which is physical exercise.

#### 3.3.2. Cluster Analysis for Lifestyle

The factors identified though FA were subject to CA in order to perceive if there was a cluster structure among the people surveyed. Cluster analysis was based on the three factors resulting from FA and started with four hierarchical methods that indicated that four clusters was the most suitable grouping structure for this set of data. Following that, the k-means method was applied using as initial solutions those obtained with the hierarchical methods. In all cases, the k-means cluster analysis produced clustering variables with means that differ significantly, as indicated by ANOVA since *p*-value < 0.0005 for the three input variables, i.e., factors F1 to f3. The results obtained for the cluster centers and number of members are presented in Table 9 and show that the four initial solutions tested converged to a similar final solution. Because the final solutions obtained from WARD and AL-BW methods converged to the exact same one, this was then considered as the final solution, which is characterized by:

- Cluster 1: individuals with strong focus on F1 and negative input for F2 and F3, i.e., those whose lifestyle is very much dominated by screens. These were named screeners;
- Custer 2: individuals with strong focus on F3 and negative input for F1 and F2, i.e., those whose lifestyle is very influenced by physical exercise. There were named exercisers;
- Cluster 3: individuals with strong focus on F2 and negative input for F1 and F3, i.e., those whose lifestyle is very strongly dominated by daily travelling hours. There were named travelers;
- Cluster 4: individuals with negative input for all three factors, i.e., those whose lifestyle is inversely associated with screens, travelling and exercise. Because these participants did not present a specific feature, this group was named others.

#### 3.3.3. Cluster Characterization

To better understand the type of people who fall into each of the four categories that cluster analysis indicated, cross tabulation between cluster membership and the sociodemographic and behavioral variables was undertaken (Table 10).

The results in Table 10 show that Portuguese participants are essentially exercisers (40.2%) while Brazilians were travelers (32.8%). While a major part of the women surveyed were exercisers (30.4%), men were mostly travelers (35.0%). Regarding the age class, young adults and the elderly were mostly screeners (44.2% and 33.3%, respectively), while middle aged adults were exercisers (36.5%), and senior adults were travelers (38.4%). While participants with a university degree were exercisers and travelers (32.8 and 33.1%, respectively) those with lower levels of education were mostly screeners (60.0% for primary school, 42.9% for basic school and 36.6% for secondary school). As for marital status, most single participants were screeners (31.7%), while those married, divorced and widowed were exercisers and travelers, in relatively similar percentages.

Table 11 shows the cross tabulation between cluster membership and some anthropometric and lifestyle variables, specifically BMI and frequency of practicing a balanced diet. Results showed that underweight people are essentially screeners (37.1%) and travelers (34.3%), those with normal weight are exercisers (31.7%) and travelers (32.4%) and so are the overweight (34.4% exercisers and 32.6% travelers). The obese are equally distributed by clusters 1 (screeners: 28.1%), 2 (exercisers: 27.2%) and 3 (travelers: 28.9%). Regarding the practice of a balanced diet, those who practice it frequently or always are exercisers and travelers, while those who never or seldom have a balanced diet are screeners or exercisers.

### 3.4. Consumption Habits Regarding Dairy Products

Table 12 shows the consumption habits of dairy products in Portugal and Brazil. Concerning milk consumption, for both countries the percentage of participants who never consume milk products is high, ranging between 46.7% (Brazil: never consume semi skimmed milk) to 94.8% (Portugal: never consume enriched milk). For those who consume milk, it was observed that 52.6% of Portuguese and 53.3% of Brazilians consume semi skimmed milk revealing a similar trend in both countries, although with variability according to the frequency of consumption. In Portugal, a higher frequency was observed for consumption of semi skimmed milk once a day (20.2%) and for Brazil for once a week (24.2%). On the other hand, the consumption of skimmed milk presents bigger differences between countries: 49.1% of Brazilians and 35.2% of Portuguese consume it, and in both countries a higher percentage of participants consume it rarely (13.8% and 27.9%, respectively, for Portugal and Brazil). This survey shows that the consumption of chocolate flavored and enriched milks is very low in these two countries, particularly in Portugal. Brazilians consume more chocolate flavored milk (34.4%) than the Portuguese (17.4%), but in both countries this is consumed with a very low frequency (seldom). In Portugal there is a slightly higher percentage of people who never consume enriched milk (94.8%) as compared with Brazil (85.3%). However, because portion and serving sizes were not specified in the questionnaires, the obtained responses may not be fully indicative, given that different participants may have interpreted the questions differently.

Milk and dairy products are considered by the FAO as important in the human diet, given their high quality protein and micronutrients in an easily absorbed form [63]. However, it has been reported in several sources that milk intake has gradually declined over the past decades [64,65,66], maybe due to the aforementioned gastrointestinal problems that can appear after lactose ingestion [14]. There are considerable studies related to the health benefits of milk consumption e.g., for bone strength [33,67,68,69], risk factor of osteoporosis [70] and protective effects against asthma, current wheeze, hay fever or allergic rhinitis, and atopic sensitization [71]. Concerning this point of view, some controversial studies can be found, like that of Wang et al. [72], which concluded that the risk associated with the consumption of milk depends on the quantities, so that moderate milk consumption diminished the risk of mortality associated with cardiovascular diseases and a high milk consumption showed an increased risk of cancer mortality. Specifically, in the two countries under study, some studies have addressed the problem of osteoporosis in Portugal and in Brazil [36,37], a recent investigation evaluated the sleep patterns in Brazilian children and the consumption of dairy products [73] and one study shows arterial hypertension management strategies according to some factors, such as dietary management, including milk and dairy product recommendations [74].

More recently a variety of enriched milks have appeared in the market, mainly with calcium and vitamin D. Calcium is critical for children’s development and is necessary for skeletal consolidation and preventing fractures and osteoporosis in old age [75,76]. According to the Dietary Guidelines Advisory Committee [77], lower calcium consumptions are linked with adverse health outcomes. The importance of vitamin D is well known owing to its importance in helping with the fixation of calcium in the bones, among other roles in the human body, namely regulating the brain, liver, lungs, heart, kidneys, skeletal, immune and reproductive systems. This vitamin also has significant anti-inflammatory, anti-aging, anti-stress, anti-arthritic, anti-osteoporosis, anti-apoptotic, wound healing, anti-cancer, anti-psychotic and anti-fibrotic actions [78,79,80,81,82,83].

As observed previously for milk consumption, a high number of participants in both countries never eat cheese (Table 12). However, this percentage is higher for Brazilians compared with the Portuguese for most types of cheese, except only for whey cheese. In fact, Portugal has a long history of eating traditional cheeses that are presently recognized with PDO (Protected Designation of Origin) [84]. The highest percentage of Portuguese who never eat a certain type of cheese is verified for imported cheeses (67.9%) and the lowest for fresh cheeses (28.8%), while for Brazilians, those who never eat cheese are, also, predominantly for imported cheeses (77.0%) and with least expression for whey cheeses (37.2%). For all categories of cheese and in both countries, the more usual consumption frequency is once a week. The differences observed in the cheese consumption patterns in both countries could be attributed to the different habits and production modes. The Brazilian cheese market has been reported to vary according to place of origin, type of milk (cow, buffalo, goat), manufacturing procedures, texture, and maturation time, among other factors [85]. For Portugal, with good pastures and a tradition in pastoralism, there are several types of cheese, made with cow, goat, sheep or mixtures of different milks, which have different tastes and consistencies. According to Guiné et al. [84], the traditional Portuguese cheeses can be classified according to the type of milk used for the cheese production, the fat content, ripening and paste consistency.

Whey is a dilute liquid resulting from cheese manufacture that contains lactose, proteins, minerals, such as calcium, and traces of fat and organic acids [84]. An important difference was observed in the consumption of whey cheese. Brazilians eat whey cheese in higher percentages (62.8%) compared to the Portuguese (54.1%), but in both cases the frequency of consumption is mostly once/week (42.9% of Portuguese and 39.1% of Brazilians).

According to Ferrão & Guiné [84], cheese is a good source of calcium, fat, protein, and some vitamins (A, B_2_ and B_12_), as well as other dietary minerals such as zinc or phosphorus. Cheese is not only consumed in its original form, and during the last decade, it has become one of the most widely used food ingredients, leading to the development of several types of low-fat cheeses that have health-promoting benefits beyond their nutritional value [86,87].

Butter is one of the most ancient and popular dairy products. This dairy product contains valuable fatty acids, as well as fat-soluble vitamins (A, D, E, K), tocopherols and carotenoids, among its important nutrients. However, the consumption of butter must be moderate because it has been linked to high cholesterol, atherosclerosis, and heart disease [88,89]. Consumer acceptance of butter is influenced by its sensory properties, which are dependent on milk raw material quality that influence the final flavor, aroma, appearance, and rheological properties [90]. Table 12 also presents the results for butter consumption in Portugal and Brazil. Around 50% of Brazilians never consume butter and the observed percentage is 43.8% for the Portuguese who also never consume butter. These percentages increase for the consumption of skimmed butter (Brazil: 82.6% never consume it; Portugal: 66.0% never consume it) and unsalted butter (Brazil: 69.1%; Portugal: 70.2%). For those who consume butter, the highest percentage of people consume it seldom, only once/week.

Yogurts are obtained from milk fermented by lactic acid bacteria such as *Lactobacillus*, *Lactococcus*, and *Leuconostoc* which allows for an extension of the product shelf life and improves its taste compared to milk, giving way to differentiated products in the market. Fermented dairy products’ consumption has been increasing widely around the world and different companies encourage new product development to satisfy the consumers with new tastes and flavors. Some of these products have demonstrated nutritional value and health benefits. For example, it has been shown that intestinal bacterial microbiota contributes to a healthy life and increases life expectancy [91,92,93,94]. This kind of product contains a high amount of live bacteria, which has benefits for human health, contributing to the maintenance and balance of the intestinal flora, facilitating digestion and preventing constipation and other gastrointestinal disorders [94,95]. Several studies state that yogurt presents antimutagenic and anticarcinogenic effects and provides protection against colorectal adenomas [95,96,97,98].

The results regarding yogurt consumption (Table 12) reveal that a high percentage of participants never consume yogurt and, on average, this value is higher for Brazilians (52.3% to 74.2% depending on the type of yogurt) when compared with the Portuguese (33.1% to 73.3%). Regarding the results in Table 12, it is observed that the classes of yogurts which are consumed by a lower percentage of participants are the ones with separated flavors (only by 26.7% of Portuguese and by 25.8% of Brazilians). Natural yogurt is consumed more frequently by the Portuguese (59.8%): 29.3% once a week and 19.3% 2–3 times/week, while the Brazilians consume 42.3% of this kind of yogurt: 30.9% once a week and 7.9% 2–3 times/week. A relatively similar trend is also observed for the aromatized yogurt. For the other classes of yogurt, the differences between Portuguese consumers and Brazilian are small.

From the collected data it was further possible to determine those participants who never consumed certain classes of dairy products or those who never consumed any of the investigated dairy products at all, and these results are shown in Table 13 for the global sample and separated by country. The results indicate that butter is the class which a highest percentage of participants never consume, 30.0%, with a higher expression in Brazil as compared to Portugal. The second class corresponds to milk products, which are never consumed by 22.8% of people, but in this case it is in Portugal that the percentage in higher. Following comes the yogurt category, never consumed by 21.1% of Brazilians and by 7.6% of Portuguese. Last appears the cheese category, with the lowest percentage of people who never consume this type of dairy product. Finally, one can see that a residual number of participants identified as never consuming any of the dairy products considered, and this result might not correspond to the reality in both countries, since this was a questionnaire survey in which the volunteers participated knowing from the start that it was about the consumption of dairy products (this information was provided before the participants gave their informed consent). Therefore, it is possible that people who never consume dairy products did not even respond to the questionnaire, by considering that their participation was not useful, or because they did not want to spend time with a subject that was not important for them.

### 3.5. Variables Influencing Dairy Product Consumption

**Figure 1:**
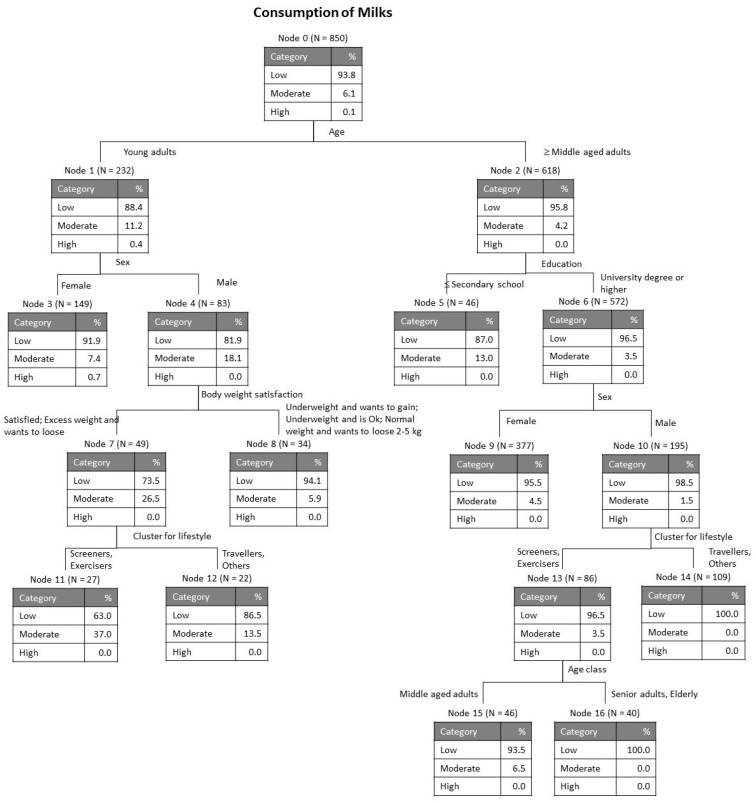
Tree classification for consumption of milks.

**Figure 2:**
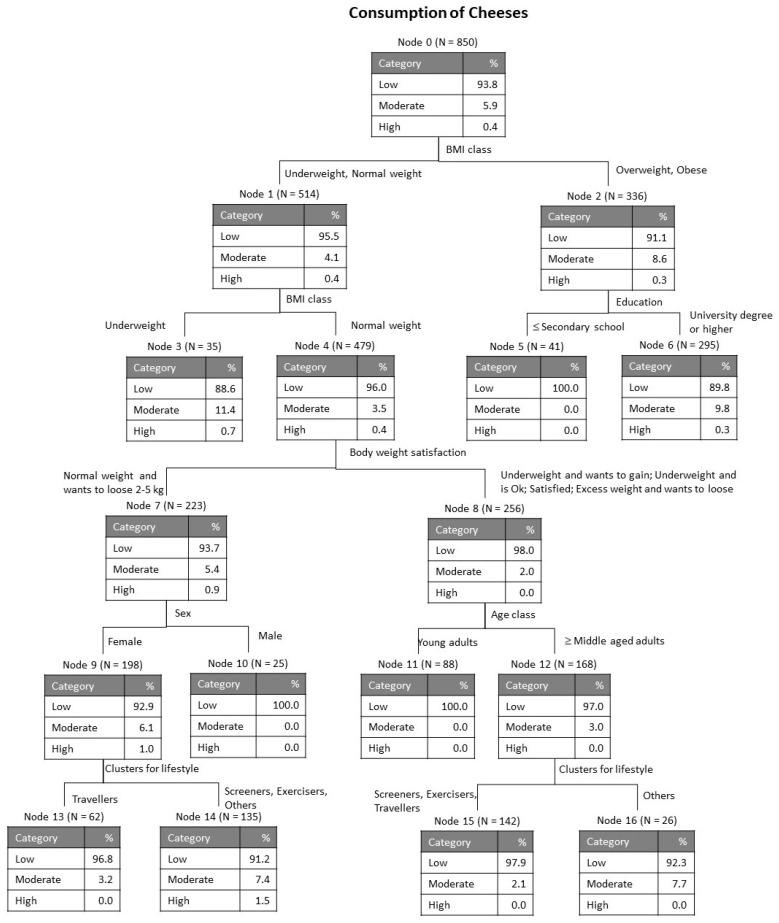
Tree classification for consumption of cheeses.

**Figure 3:**
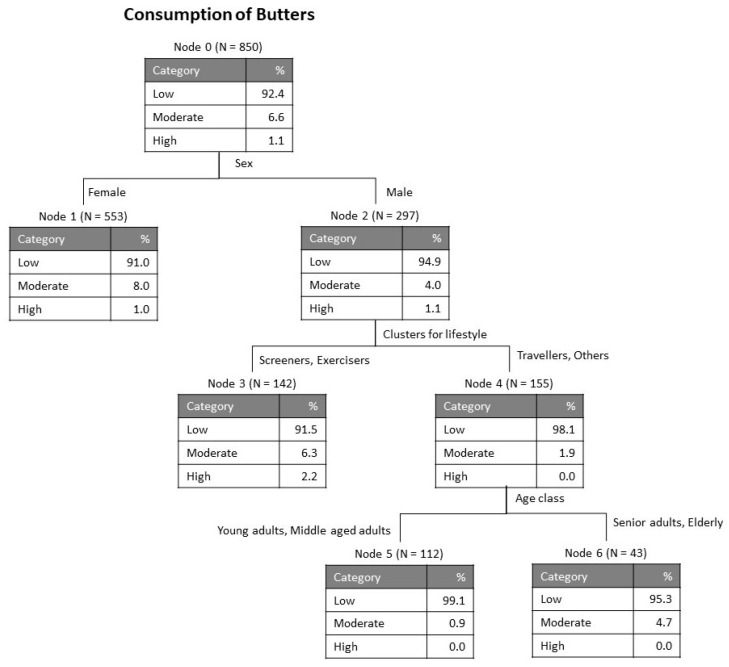
Tree classification for consumption of butters.

**Figure 4:**
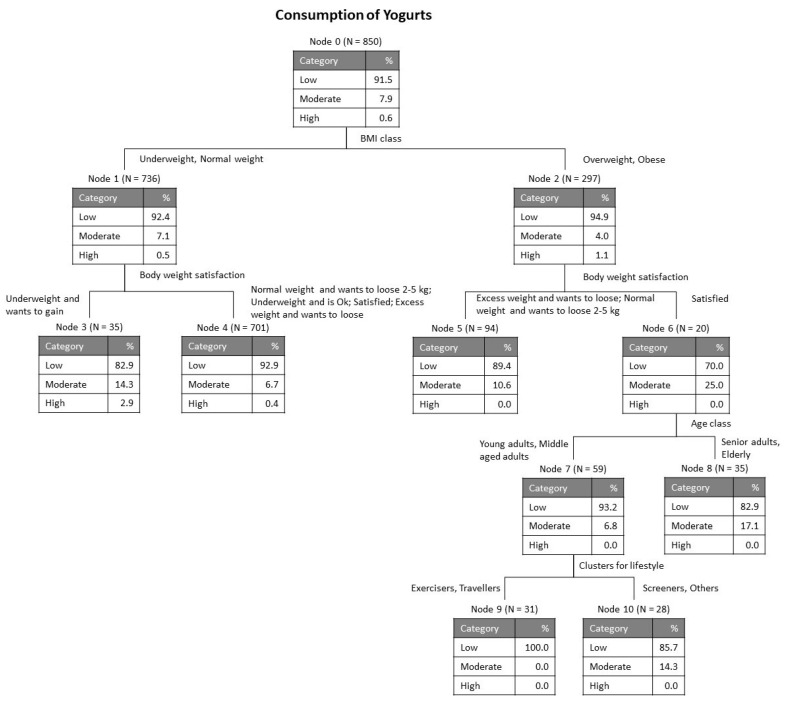
Tree classification for consumption of yogurts.

As explained in the section Materials and Methods, the variables accounting for the average level of consumption of the four categories of dairy products (milk, cheese, butter and yogurt) were submitted to a tree classification analysis for evaluation of the relative importance of each of the possible influential variables considered: country, sex, age class, education, BMI class, satisfaction with body weight, balanced diet and lifestyle clusters. Figure 1, Figure 2, Figure 3 and Figure 4 show the obtained classification trees, and they reveal that some of the variables considered in the analysis were not influential, for example, variables such as country and balanced diet never appeared in any of the diagrams, meaning that they do not determine the consumption of any of the dairy products evaluated.

The tree in Figure 1, for consumption of milk products, contains 4 levels and 17 nodes, of which 9 are terminal. The risk estimate for re-substitution was 0.062 with standard error 0.008 and the risk estimate for cross-validation was 0.061 with standard error 0.008. These values indicate goodness of fit to the model. The results in Figure 1 reveal that, for the whole sample (node 0), a huge majority of participants have a low milk consumption (93.8%) and that the first discriminant variable was age, so that younger people (from 18 to 30 years) tend to have a slightly higher consumption of milk than older people (moderate consumption: 11.2% and 4.2%, respectively, for people up to 30 years and older). For young people, the second discriminating factor was sex, with men showing higher consumption of milk as compared with women. For young men, the next discriminant variable was body weight satisfaction and the final differentiating factor was lifestyle cluster. Regarding older people, the next discriminating factor after age was education, with lower milk consumption for people with university degrees or higher levels of education. Following in the order of appearance the discriminating variables were sex, lifestyle cluster and age class again, differentiating in the last level middle aged adults (93.5% low and 6.5% moderate consumption) from senior adults and elderly (100% low consumption).

The tree in Figure 2 for the consumption of cheese has 5 levels with 17 nodes, including 9 terminals. The risk estimates for re-substitution and for cross-validation were in both cases 0.062 with standard error 0.008. The results for the whole set of participants (node 0) indicate that, similarly to milk consumption, cheese consumption is also very low (93.8% low, and only 5.9% moderate). The discriminant variable in the first level was BMI class, separating the overweight and obese participants as having slightly higher consumption of cheese (8.6% moderate and 91.1% low). For these, the next discriminant variable was education and in this case those with higher levels of education tend to have a higher consumption of cheese (8.6% moderate against 0% for those with lower education). For the branch of underweight and normal weight, BMI class was again the differentiating factor, with lower consumption for normal weight participants (3.5% moderate against 11.4% moderate for the underweight). The following discriminant variables were body weight satisfaction (level 3), sex and age class (level 4) and lifestyle cluster (level 5).

Figure 3 presents the results of the tree obtained for butter consumption. This has only 3 levels, representing 7 nodes, of which 4 were terminal. The risk parameters were equal for re-substitution and cross-validation: risk estimate = 0.076, standard error = 0.009. In the case of butter, the values for the whole sample are low (92.4% low, 6.6% for moderate and 1.1% for high consumption), being in line with the trends previously observed for milk and for cheese. The first discriminant was sex, differentiating men as having lower consumption than women (94.9% and 91.0%, respectively). For women the node was terminal, while for the men, the following discriminant was lifestyle cluster, separating the screeners and exercisers as showing a slightly higher butter consumption (6.3% moderate as compared with 1.9% moderate for travelers and others). Finally, at level 3 the discriminating variable was age class, separating people over 50 years, which presented a higher butter consumption (4.7% moderate).

Figure 4 shows the tree for yogurt consumption, with 4 levels and 11 nodes (six of which are terminal). The risk estimate for re-substitution was 0.085 with standard error 0.010 and equal values were obtained for cross-validation. The consumption of yogurts is again low (91.5% at node 0). The first discriminant was BMI, as was observed for cheese, and the discriminant at level 2 was body weight satisfaction, regardless of the BMI class (i.e., on both branches). For the overweigh and obese (i.e., BMI of 25 or over) who are satisfied with their body weight, 25% have a moderate yogurt consumption. For these, the next level was separated according to their age class, and for the participants aged up to 50 years the last discriminant was lifestyle cluster.

Overall, these results seem to indicate that the most relevant discriminant factors for dairy consumption were age, BMI, sex, education, satisfaction with body weight and lifestyle cluster, following more or less the same order regardless of the type of dairy product. On the contrary, factors such as country or balanced diet were found to have no discriminant capacity for the variables under study, i.e., the consumption of dairy products is not influenced significantly by these two variables.

According to Wolf et al. [99,100] age is a factor determining milk consumption in the United States, with people born in the 1990s consuming milk less often than earlier generations, and this trend is expected to continue with the replacement of older generations by younger ones. Nevertheless, the consumption of other dairy products seems to be increasing in the US, in the case of cheese mostly because it is widely used in pizzas and in the case of butter because there has been a setback regarding health views of butterfat [101]. The study by Xu et al. [4] highlighted also differences in dairy consumption according to sex and BMI. On the other hand, lifestyle behaviors have been proven to influence dairy consumption according to the recent study by Santaliestra-Pasías et al. [102]. Their results suggest that European children with healthier lifestyles, specifically regarding aspects such as physical activity and sedentary behaviors, tend to consume higher quantities of milk and yogurt.

## 4. Limitations of the Study

Although providing important insights into the consumption habits of the Portuguese and Brazilian citizens, this study has some limitations, that are worth mentioning. One of these limitations is related to the sample sizes. Although it was possible to recruit a similar number of participants from the two countries involved, the size of Portugal and Brazil are unequal, and therefore it could be beneficial to have samples proportional to the population of each of the countries. Also, the group sizes for the sociodemographic variables considered are not equal, for example there were more women than men and a lower number of older participants or with lower levels of education. However, due to financial restrictions it was not possible to organize another type of data collection. One other limitation can be associated with the period of data collection. Although the data collection was mostly done in the period pre covid-19 outbreak, in Portugal the outbreak was felt after mid-March, but in Brazil the most critical period was later and therefore this outbreak may have had some influence only on data collected in Portugal in the last month. Still, in Portugal during the confinement period, no shortage whatsoever in the food supply was observed, and people were always allowed to go out for food shopping, so the possible influences are expected to be very mild. Another factor is related to possible loss of jobs and consequent reduction in the available monthly budget, which could influence people’s food purchases. However, in the early days of the confinement the social measures implemented prevented a mass loss of jobs and therefore this problem could have had a higher importance if the data collection was extended beyond April, which it was not.

Other limitations relate to some additional aspects of the research that could in future studies be addressed, such as for example the motivations that drive consumers in relation to the consumption or non-consumption of dairy products. This would also be an interesting aspect to explore in the future, to better understand the reasons why people consume or avoid different dairy products. Finally, it would also be interesting to replicate this investigation in other countries to see if the observed low influence of the country on the dairy products’ pattern of consumption would still be maintained, or if these similarities observed are because the two countries involved share a common culture and history due to the colonization of Brazil by the Portuguese.

## 5. Conclusions

The present work established some relevant facts about the consumption of dairy products in two counties, one situated in Europe and the other in Latin America. Although some small differences were observed in the consumption patterns in both countries, a worrying fact is that the levels of consumption for all dairy products studied were frankly low. Regarding milk consumption, semi skimmed milk is never consumed by about half of the participants, those who never consume skimmed milk are even more in number, and these numbers increase again for chocolate flavored and enriched milks. Cheeses are also consumed by only small parts of the population in these two countries, with the least consumed being imported cheeses in both countries followed by soft paste cheeses in Brazil and Portugal and whey cheese in Portugal. For those who consume cheese, they do so with a low frequency: once a week or sometimes (2–3 times per week). Butter is also consumed by only about half of the adult population, but the percentage of those who never consume butter increases for skimmed butter and unsalted butter, these last being least preferred when compared to regular milk butter. Yogurt consumption follows the same low consumption trends of other dairy products. In order of preference, the most frequently consumed yogurt types are liquid yogurts and natural yogurts in Portugal and creamy fruit pulp yogurt, liquid yogurt and Greek type yogurt in Brazil.

This work further studied some anthropometric facts of the surveyed sample as well as behavioral aspects, allowing the establishment of groups according to lifestyles, as determined by cluster analysis. Four clusters were identified: 1—Screeners, 2—Exercisers, 3—Travelers and 4—Others. The screeners were mostly single young adults, with low education and from Brazil. The Exercisers were mostly women with ages comprised between 30 and 65 years, with a university degree and from Portugal. The Travelers were mostly men aged between 30 and 65 years, also with a university degree and divorced or widowed. These lifestyles were found to be influential to the consumption of dairy products for all classes of dairy tested: milk, cheese, yogurt and butter. Additionally, other influential factors found were age, sex, education, BMI and satisfaction with body weight. The influence of country was not a meaningful discriminant, in relation to the other variables included in the classification analysis. This might be due to the cultural similarity between the two countries studied, which, although being in different parts of the globe, have a historic and cultural common past. For these reasons it would be interesting to replicate this study in the future in other countries to evaluate the extension of possible country variability in the consumption of dairy products.

## Figures and Tables

**Table 1:** Sociodemographic characteristics of the participants.

Variable	Class	N	%
*Country*	Brazil	430	50.6
	Portugal	420	49.4
*Sex*	Female	553	65.1
	Male	297	34.9
*Age Class*	Young adults (18 ≤ age ≤ 25 years)	231	27.2
	Middle aged adults (26 ≤ age ≤ 50 years)	403	47.5
	Senior adults (51 ≤ age ≤ 65 years)	203	23.9
	Elderly (age ≥ 66 years)	12	1.4
*Marital status*	Single	372	43.8
	Married or living together	412	48.5
	Divorced or separated	53	6.2
	Widowed	13	1.5
*Education level*	Primary school (4 years)	5	0.6
	Basic school (9 years)	7	0.8
	Secondary school (12 years)	101	11.9
	University degree	737	86.7

**Table 2:** Anthropometric measures of the participants.

*Sex*	*Measure ^(a)^*	*N ^(b)^*	*Minimum*	*Maximum*	*M ± SD ^(c)^*
Women	Height (m)	552	1.38	1.80	1.63 ± 0.06
	Weight (kg)	552	40.00	120.00	63.71 ± 11.96
	BMI (kg/m^2^)	552	15.43	43.03	23.86 ± 4.31
Men	Height (m)	297	1.54	2.00	1.76 ± 0.07
	Weight (kg)	297	44.0	140.0	81.87 ± 15.28
	BMI (kg/m^2^)	297	15.94	41.67	26.34 ± 4.40
Global	Height (m)	849	1.38	2.00	1.68 ± 0.09
	Weight (kg)	849	40.0	140.00	70.06 ± 15.80
	BMI (kg/m^2^)	849	15.43	43.03	24.73 ± 4.50

^(a)^ BMI = body mass index = (weight/height^2^). ^(b)^ N = number of values used in the calculations. ^(c)^ Mean values (M) and standard deviation (SD).

**Table 3:** Classification of the participants’ Body Mass Index (BMI = weight/height^2^).

*BMI ^1^ Classification:*	*Women*		*Men*		*Global*	
	N	%	N	%	N	%
Underweight: BMI < 18.5	29	5.3	6	2.0	35	4.1
Normal weight: 18.5 ≤ BMI < 25	359	65.0	120	40.4	479	56.5
Overweight: 25 ≤ BMI < 30	105	19.0	116	39.1	221	26.0
Obese: BMI ≥ 30	59	10.7	55	18.5	114	13.4

^1^ BMI = body mass index = (weight/height^2^).

**Table 4:** Practice of a balanced diet.

Variable/Group		Never	Seldom	Sometimes	Frequently	Always
Global (%)		0.9	5.6	30.0	46.9	16.5
Sex	Women (%)	0.4	5.1	28.9	47.9	17.7
	Men (%)	2.0	6.8	32.0	45.1	14.1
	χ^2^ test *	N = 850; *p*-value = 0.060				
Country	Brazil (%)	1.2	8.4	35.8	41.6	13.0
	Portugal (%)	0.7	2.9	24.0	52.4	20.0
	χ^2^ test *	N = 850; *p*-value < 0.0005; Cramer’s V = 0.198				
BMI class	Underweight	0.0	5.7	45.7	37.1	11.5
	Normal weight	0.4	4.2	27.3	47.8	20.3
	Overweight	0.9	6.8	29.4	50.7	12.2
	Obese	3.5	9.6	37.7	38.6	10.6
	χ^2^ test *	N = 849; *p*-value = 0.001; Cramer´s V = 0.116				
Age class	Young adults	1.7	8.7	41.6	35.0	13.0
	Middle aged adults	0.7	5.5	25.3	53.8	14.9
	Senior adults	0.5	3.0	26.6	48.3	21.6
	Elderly	0.0	0.0	25.0	16.7	58.3
	χ^2^ test *	N = 849; *p*-value < 0.0005; Cramer´s V = 0.148				
Marital status	Single	1.3	7.0	36.3	43.8	11.6
	Married	0.5	5.1	24.5	49.3	20.6
	Divorced	1.9	1.9	30.2	47.2	18.8
	Widowed	0.0	0.0	23.1	61.5	15.4
	χ^2^ test *	N = 850; *p*-value = 0.009; Cramer´s V = 0.102				
Education	Primary	20.0	0.0	60.0	20.0	0.0
	Basic	0.0	14.3	57.1	14.3	14.3
	Secondary	0.0	11.9	32.7	44.6	10.8
	University	0.9	4.7	29.2	47.8	17.3
	χ^2^ test *	N = 850; *p*-value < 0.0005; Cramer´s V = 0.123				

* Chi-square test, level of significance of 5% (*p* < 0.05). The Cramer´s V was only indicated for cases where significant differences were observed.

**Table 5:** Satisfaction with body weight.

**Satisfaction**	**Global**	**BMI Class**			
		**Underweight**	**Normal weight**	**Overweight**	**Obese**
I am satisfied with my body weight and I want to keep it (%)	25.5	22.9	39.5	7.2	3.5
I have normal weight, but would like to lose 2 to 5 kg (%)	40.1	5.7	46.4	48.9	7.0
I am underweight, but I feel good like this (%)	2.5	25.7	2.5	0.0	0.0
I am underweight and would like to gain 2 to 5 kg (%)	4.2	42.9	4.2	0.0	0.0
I am overweight and I would like to lose a few kilos, but I have tried and I cannot (%)	20.9	2.8	4.2	32.1	75.4
I am overweight and do nothing to change this (%)	0.0	0.0	0.0	0.0	0.0
Other (%)	6.8	0.0	3.2	11.8	14.1
χ^2^ test * (for BMI Class)		N = 849; *p*-value < 0.0005; Cramer´s V = 0.500			
**Satisfaction**	**Balanced diet**				
	**Never**	**Seldom**	**Sometimes**	**Frequently**	**Always**
I am satisfied with my body weight and I want to keep it (%)	12.5	14.6	17.6	26.6	41.4
I have normal weight, but would like to lose 2 to 5 kg (%)	12.5	29.2	37.3	45.4	35.7
I am underweight, but I feel good like this (%)	12.5	6.3	4.7	1.0	0.7
I am underweight and would like to gain 2 to 5 kg (%)	0.0	4.2	6.7	3.3	2.1
I am overweight and I would like to lose a few kilos, but I have tried and I cannot (%)	25.0	45.7	26.7	16.5	14.4
I am overweight and do nothing to change this (%)	0.0	0.0	0.0	0.0	0.0
Other (%)	37.5	0.0	7.1	7.2	5.7
χ^2^ test * (for balanced diet)	N = 850; *p*-value < 0.0005; Cramer´s V = 0.165				

* Chi-square test, level of significance of 5% (*p* < 0.05). The Cramer´s V was only indicated for cases where significant differences were observed.

**Table 6:** Physical activity.

Intensity of Physical Activity		N	%
Physical exercise	Never	148	17.4
	Occasionally (1 time/week)	330	38.8
	Moderate (2–3 times/week)	288	33.9
	Intense (>3 times/week)	84	9.9
Daily time walking or riding a bike	0:00–0:30 h	444	52.2
	0:30–1:00 h	287	33.8
	1:00–2:00 h	85	10.0
	2:00–5:00 h	26	3.1
	>5:00 h	8	0.9

**Table 7:** Sedentary lifestyle.

Inactivity		Hours per day				
		0:00–0:30	0:30–1:00	1:00–2:00	2:00–5:00	>5:00
Watching TV	N	193	246	275	117	19
	%	22.7	28.9	32.4	13.8	2.2
Screen entertaining ^1^	N	339	189	194	98	30
	%	39.9	22.2	22.8	11.5	3.6
Working on computer	N	65	70	121	258	336
	%	7.6	8.2	14.2	30.4	39.6
Traveling inactive ^2^	N	461	258	104	19	8
	%	54.2	30.4	12.2	2.2	1.0

^1^ Playing with computer or mobile phone or on social networks. ^2^ In car, motorbike, bus, train, etc.

**Table 8:** Component matrix obtained by factor analysis to variables related with active or sedentary lifestyles.

Factors	Variable	Component
F1: Screens	TV = daily hours watching TV	0.675
	LC = daily hours leisure: playing with computer or mobile phone or on social networks	0.722
	WC = daily hours working on computer	−0.558
F2: Travelling	TI = daily hours traveling inactive (in car, motorbike, bus, train, etc.)	0.727
	WR = daily hours traveling walking or riding a bike	0.739
F3: Exercise	PE = practice of physical exercise	0.904

**Table 9:** Final cluster centers and number of members.

Cluster	Hierarchical Initial Solution	Number of Members	Final Cluster Centers		
			Factor F1	Factor F2	Factor F3
Cluster C1	Ward	179	1.390	−0.167	−0.451
	AL-WG	179	1.390	−0.167	−0.451
	AL-BG	177	1.351	−0.229	−0.465
	Centroid	178	1.364	−0.211	−0.440
Cluster C2	Ward	272	−0.168	−0.285	1.009
	AL-WG	272	−0.168	−0.285	1.009
	AL-BG	284	−0.238	−0.386	0.922
	Centroid	281	−0.202	−0.321	0.964
Cluster C3	Ward	132	−0.261	1.697	−0.083
	AL-WG	132	−0.261	1.697	−0.083
	AL-BG	140	−0.048	1.681	0.114
	Centroid	135	−0.156	1.720	−0.033
Cluster C4	Ward	267	−0.632	−0.437	−0.685
	AL-WG	267	−0.632	−0.437	−0.685
	AL-BG	249	−0.662	−0.342	−0.785
	Centroid	256	−0.645	−0.407	−0.735

**Table 10:** Cluster membership according to sociodemographic variables.

Variable	Group	Cluster 1 Screeners	Cluster 2 Exercisers	Cluster 3 Travelers	Cluster 4 Others
Country	Brazil (%)	30.0	22.8	32.8	14.4
	Portugal (%)	11.9	40.2	31.2	16.7
Sex	Women (%)	20.8	34.2	30.4	14.6
	Men (%)	21.5	26.3	35.0	17.2
Age	Young adults (%)	44.2	19.9	24.2	11.7
	Middle aged adults (%)	11.4	36.5	33.5	18.6
	Senior adults (%)	12.8	35.0	38.4	13.8
	Elderly (%)	33.3	25.0	25.0	16.7
Education level	Primary (%)	60.0	0.0	0.0	40.0
	Basic (%)	42.9	28.6	0.0	28.6
	Secondary (%)	36.6	22.8	27.7	12.9
	University (%)	18.5	32.8	33.1	15.6
Marital status	Single (%)	31.7	24.5	28.5	15.3
	Married (%)	12.9	37.4	34.2	15.5
	Divorced (%)	11.3	32.1	37.7	18.9
	Widowed (%)	15.4	38.5	38.5	7.7

**Table 11:** Cluster membership according to some anthropometric and lifestyle variables.

Variable	Group	Cluster 1 Screeners	Cluster 2 Exercisers	Cluster 3 Travelers	Cluster 4 Others
IMC class	Underweight (%)	37.1	20.0	34.3	8.6
	Normal weight (%)	20.9	31.7	32.4	15.0
	Overweight (%)	15.4	34.4	32.6	17.6
	Obese (%)	28.1	27.2	28.9	15.8
Balanced diet	Never (%)	37.5	37.5	0.0	25.0
	Seldom (%)	37.5	37.5	12.5	12.5
	Sometimes (%)	32.9	29.4	20.0	17.6
	Frequently (%)	14.8	32.1	40.9	12.3
	Always (%)	10.7	30.7	37.1	21.4

**Table 12:** Frequency of consumption of dairy products in Portugal and Brazil.

Product	Country ^1^	Frequency of Responses ^2^ (%)				
		Never	Seldom	Sometimes	Frequently	Always
Category: Milk						
Semi skimmed	Portugal	47.4	17.4	11.0	20.2	4.0
	Brazil	46.7	24.2	14.2	9.1	5.8
Skimmed milk	Portugal	64.8	13.8	7.1	9.5	4.8
	Brazil	50.9	27.9	9.3	6.7	5.1
Chocolate flavored	Portugal	82.6	11.2	4.3	0.7	1.2
	Brazil	65.6	24.0	6.7	3.3	0.5
Enriched milk	Portugal	94.8	2.9	1.7	0.7	0.0
	Brazil	85.3	10.7	2.8	0.7	0.5
Category: Cheese						
Soft paste cheese	Portugal	43.3	44.0	9.8	2.6	0.2
	Brazil	70.7	21.2	6.0	1.4	0.7
Cured hard paste cheese	Portugal	45.2	38.3	12.9	3.3	0.2
	Brazil	57.7	29.3	10.2	2.1	0.7
Fresh cheese	Portugal	28.8	46.4	18.3	5.7	0.7
	Brazil	41.4	38.8	14.0	4.4	1.4
Whey cheese	Portugal	46.9	42.9	7.1	2.9	0.2
	Brazil	37.2	39.1	15.6	6.3	1.9
Semi skimmed sliced cheese	Portugal	35.0	31.0	24.0	8.1	1.9
	Brazil	39.3	30.7	21.2	6.5	2.3
Skimmed sliced cheese	Portugal	35.0	31.0	24.0	8.1	1.9
	Brazil	39.3	30.7	21.2	6.5	2.3
Imported cheeses	Portugal	67.9	28.1	3.8	0.2	0.0
	Brazil	77.0	19.1	3.3	0.5	0.2
Category: Butter						
Milk butter	Portugal	43.8	22.9	17.1	11.0	5.2
	Brazil	49.5	24.0	14.7	6.3	5.6
Skimmed butter	Portugal	66.0	19.5	8.3	5.0	1.2
	Brazil	82.6	14.0	3.5	0.0	0.0
Butter without salt	Portugal	70.2	20.5	4.8	3.1	1.4
	Brazil	69.1	20.0	6.7	3.5	0.7
Category: Yogurt						
Natural yogurt	Portugal	40.2	29.3	19.3	8.8	2.4
	Brazil	57.7	30.9	7.9	2.3	1.2
Aromatized yogurt	Portugal	42.1	33.3	20.0	3.3	1.2
	Brazil	62.1	25.6	8.6	2.6	1.2
Creamy fruit pulp yogurt	Portugal	60.5	25.7	11.0	2.1	0.7
	Brazil	55.6	30.0	9.8	3.7	0.9
Yogurt with fruit pieces	Portugal	61.4	23.8	11.4	2.1	1.2
	Brazil	65.1	26.5	5.3	2.3	0.7
Liquid yogurt	Portugal	33.1	32.1	20.5	11.2	3.1
	Brazil	52.3	34.0	9.5	3.5	0.7
With separated flavors	Portugal	73.3	20.5	4.3	1.7	0.2
	Brazil	74.2	18.4	5.1	2.1	0.2
Greek type yogurt	Portugal	50.0	33.3	11.7	4.0	1.0
	Brazil	59.8	30.0	5.8	3.5	0.9

^1^ Number of participants: N(Portugal) = 420, N(Brazil) = 430. ^2^ Frequency: Seldom = once/week, Sometimes = 2 to 3 times/week, Frequently = once/day, Always = more than once/day.

**Table 13:** Participants who “never” consume dairy products in Portugal and Brazil.

	Portugal (N = 420)		Brazil (N = 430)		Global (N = 850)	
	N	%	N	%	N	%
Participants who ”never” eat dairy products in the category Milk	121	28.8	73	17.0	194	22.8
Participants who ”never” eat dairy products in the category Cheese	36	8.6	26	6.0	62	7.3
Participants who ”never” eat dairy products in the category Butter	100	23.8	155	36.0	255	30.0
Participants who ”never” eat dairy products in the category Yogurt	32	7.6	91	21.1	123	14.4
Participants who ”never” eat dairy products in any of the categories	5	1.2	5	1.2	10	1.2

## Appendix A

The questions included in the questionnaire were distributed by tree different sections as follows:

**Part 1—Sociodemographic data**

**Country:** Portugal □_1_  Brazil □_2_

**1. Age:** _______ years

**2. Sex:** Female □_1_ Male □_2_

**3. Level of education:**

Primary (4 school years) □_1_    Basic school (9 years) □_2_    Secondary school (12 years) □_3_

University degree or higher □_4_

**4. Marital status:**

Single □_1_    Married □_2_    Divorced □_3_    Widowed □_4_

**5. Profession:**

Student □_1_    Employee for the government □_2_    Employee for private companies □_3_

Self-employed freelance □_4_    Businessmen □_5_    Unemployed□_6_    Other□_7_

**Part 2—Anthropometric data and behavioural aspects**

**6. Height:** __________ meters

**7. Weight:** ___________ kg

**8. Do you practice physical exercise?**

Never □_1_   Occasional (once/week) □_2_   Moderate (2–3 times/week) □_3_   Intense (+ 3 times/week) □_4_

**9. Daily hours watching TV:**

0–30 min □_1_    30 min–1 hour □_2_    1–2 hour □_3_    2–5 hours □_4_    More than 5 hours □_5_

**10. Daily time playing on computer or mobile phone or on social networks:**

0–30 min □_1_    30 min–1 hour □_2_   1–2 hour □_3_    2–5 hours □_4_    More than 5 hours □_5_

**11. Daily time working on the computer:**

0–30 min □_1_    30 min–1 hour □_2_   1–2 hour □_3_    2–5 hours □_4_    More than 5 hours □_5_

**12. Daily time traveling sedentary (car, motorcycle, train, bus, etc…):**

0–30 min □_1_    30 min–1 hour □_2_    1–2 hour □_3_    2–5 hours □_4_    More than 5 hours □_5_

**13. Daily walking time (on foot, by bicycle,…):**

0–30 min □_1_    30 min–1 hour □_2_    1–2 hour □_3_    2–5 hours □_4_    More than 5 hours □_5_

**14. Do you believe that you have a balanced diet?**

Never □_1_    Seldom □_2_    Sometimes □_3_    Several times/week □_4_    Always □_5_

**15. Are you satisfied with your bodyweight?**

I am satisfied with my weight and wish to keep it □_1_

I am of normal weight, but I would like to lose 2 to 5 kg □_2_

I am underweight, but I feel good □_3_

I am underweight and would like to gain 2 to 5 kg □_4_

I am overweight and I would like to lose a few pounds, but I have tried and I cannot □_5_

I am overweight and do nothing to change □_6_

Other □_7_

**Part 3—Consumption habits regarding dairy products**

**16. How often do you consume the following dairy products?**

**Product**	**Never**	**Seldom** ***Once/week***	**Sometimes** ***2–3 x/week***	**Frequently** ***once/day***	**Always** ***+ 1 x/day***
**Category: Milk**					
(1) Semi skimmed milk					
(2) Skimmed milk					
(3) Chocolate flavored milk					
(4) Enriched milk					
**Category: Cheese**					
(5) Soft paste cheese					
(6) Cured hard paste cheese					
(7) Fresh cheese					
(8) Whey cheese					
(9) Semi skimmed sliced cheese					
(10) Skimmed sliced cheese					
(11) Imported cheeses					
**Category: Butter**					
(12) Milk butter (not vegetable spreads)					
(13) Skimmed butter					
(14) Butter without salt					
**Category: Yogurt**					
(15) Natural yogurt					
(16) Aromatized yogurt					
(17) Creamy fruit pulp yogurt					
(18) Yogurt with fruit pieces					
(19) Liquid yogurt					
(20) Yogurt with separated flavors (cereals, jam, chocolate chips, etc.)					
(21) Greek type yogurt					
